# Excerpts

**Published:** 1889-12

**Authors:** John A. Miller

**Affiliations:** Niagara and Cornell Universities


### EXCERPTS.

By JOHN A. MILLER, Ph. D., M. D., of Niagara and Cornell Universities.

INFLUENCE OF SACCHARIN ON DIGESTION—BY STIFT (*Bied. Centr.*, xviii., p. 458).

It is well known that saccharin is not capable of being digested, that it passes unchanged through the organism. In experiments made by the author on himself, in which 46.2 grains of saccharin a day was taken, a slight *purgative* action was observed, followed by a loss of appetite. In order to ascertain what action, if any, saccharin had upon the digestive ferments, experiments were made with meat, egg-albumin, casein, and pea-meal, with and without the addition of saccharin. From these experiments the author ascertained that the presence of saccharin delayed the solution of the albuminoids, which was not complete after twelve hours contact with the gastric juice. The greater the amount of saccharin present, the more marked was its action. Similar results were obtained with diastase and with pancreatic ferment. Saccharin, therefore, interferes with digestion, and must be considered injurious to health.

ACTION OF CARBOHYDRATES ON THE ANIMAL ORGANS—BY P. ALBERTONI (*Chem. Centr.*, 1889, page 608).

Experiments which were made on man and on lower animals to determine the action of glucose, maltose and saccharose on the animal economy, showed that their absorption into the blood is attended with an increased action of the pulse. In the dog the increase being from 15-20 beats per minute, and in man from 6-8 beats per minute. The pressure of the blood is also increased by about 15-20 m. m. of mercury, and an increased action of the heart being also observed. The blood-vessels are enlarged, and an increased quantity of blood, almost double the normal, flows from them. The rate of circulation is also much increased. Morphine and chloral prevent the action of the carbohydrates on the circulation.

From these experiments the author draws the conclusion that the carbohydrates are not to be regarded as a food proper, but that they have an important effect on the action of the heart. [Whether or not the carbohydrates are to be considered as foods, depends entirely upon our definition of that term. If foods are only such substances as can replace body waste, or can be used in building up the tissue of the body, then carbohydrates can hardly be called foods. We must not forget, however, that there are other elements, fully as important as the replacing of broken-down tissue, to be considered in the study of vital phenomena. For example, the temperature of the body is as important as the building up of tissue, and each must be considered in its proper place when studying the foods in their relation to the animal economy. The increased rate of circulation, which means an increase in temperature to at least a slight degree, as well as the number of caloric units evolved during the combustion of the carbohydrates, are simply means of sustaining the proper temperature of the body.—J. A. M.]

PTOMAINES IN CYSTINURIA—BY L. V. UDRÁNSZKY AND E. BAUMANN (*Ziet. Physiol. Chem.*, xiii., p. 562).

The authors submitted the urine of a patient suffering with cystin calculi, to an examination for the purpose of determining whether or not ptomaines were present. They succeeded in isolating the diamines (ptomaines), cadaverine ($C_5H_{14}N_2$), and putrescine ($C_4H_{12}N_2$). Of these two alkaloidal substances, cadaverine was the more abundant. The quantity per day varied very much; on one occasion 3.19 grains of cadaverine and 1.69 grains of putrescine, while on another occasion only the merest traces were obtained.

The diamines are absent both in normal urine and feces, and also in cases of acute and chronic cystitis, as well as in the urine of patients suffering from scarlatina, diphtheria, typhoid pneumonia, peritonitis, and other suppurative processes.

In the two cases of cystinuria, reported by Brieger and Stadthagen, both cadaverine and putrescine were present in the urine.

The investigations of Brieger show that diamines are formed in certain putrefactive process, especially in cultivations of cholera-bacillus and the Finkler-Prior vibrio.

There is one circumstance, then, which cholera and cystinuria have in common,—the formation of diamines; and there is but little doubt that in both cases they are formed by the agency of microörganisms.

In the case under investigation, the authors not only found diamines present in the urine, but succeeded in isolating cadaverine and putrescine from the feces of the patient; putrescine being the more abundant of the two.

Should later investigations show that these diamines are a constant formation in the alimentary canal of patients suffering from cystinuria, the ultimate conclusion must needs be that cystinuria is an infectious disease. The microörganisms, however, which are present, would differ from most pathogenic bacteria in their prolonged existence in the same individual.

[According to the observations of Grawitz, cadaverine seems to hinder the growth of bacteria. This fact, if true, might account for their long existence as mentioned.—J. A. M.]

Relation of Ptomaines to Infectious Fevers—By A. P. Luff (*Brit. Med. Jour.*, 2, 1889, p. 193).

The author simply reports one case of typhoid fever and several cases of scarlet fever, in which small quantities of alkaloids of a doubtful nature were obtained from the urine of these patients.

Proteid Poisons—By S. Martin (*Brit. Med. Jour.*, 1889).

The author investigated jequirity (Abrus precatorious) a substance employed in ophthalmic surgery to produce inflammation of the conjunctiva. He found that the poisonous properties of this plant lay in the two proteids which it contains.

These two proteids both produce nearly the same effects, *i. e.*, local edema and ecchymosis at the seat of inoculation, with ecchymoses in the serous membranes, and gastro-enteritis, the blood in many cases remaining fluid. There is a gradual sleepiness, which ends in coma, followed after death by a rapid onset of rigor mortis. Both proteids have a remarkable lowering effect on the body temperature. Temperatures above eighty degrees destroy their activity.

---

The Astley Cooper Prize, amounting to $1,500, will be awarded in 1892. The question proposed is, The Influence of Microörganisms upon Inflammation. The papers must be written in English, or accompanied by an English translation, and should be addressed before January 1, 1892, to Guy's Hospital, London. The prize will not be awarded to two or three working conjointly.—*Times and Register.*

